# The SagA of *E. faecium*

**DOI:** 10.7554/eLife.97277

**Published:** 2024-04-05

**Authors:** Rishika Prasad, Robert R Jenq

**Affiliations:** 1 Department of Genomic Medicine, Division of Cancer Medicine, The University of Texas MD Anderson Cancer Center Houston United States

**Keywords:** *Enterococcus faecium*, peptidoglycan hydrolase, immunotherapy, SagA, cancer therapy, microbiome, Other

## Abstract

An enzyme that remodels the cell wall of *Enterococcus faecium* helps these gut bacteria to divide and generate peptide fragments that enhance the immune response against cancer.

**Related research article** Klupt S, Fam KT, Zhang X, Chodisetti PK, Mehmood A, Boyd T, Grotjahn D, Park D, Hang HC. 2024. Secreted antigen A peptidoglycan hydrolase is essential for *Enterococcus faecium* cell separation and priming of immune checkpoint inhibitor cancer therapy. *eLife* **13**:RP95297. doi: 10.7554/eLife.95297.

Bacteria and other microbes residing in our intestinal tract – known as gut microbiota – are important for maintaining health and normal immunity. Among these is a group of lactic acid-producing bacteria called *Enterococcus* that inhibit the growth of harmful pathogens and aid digestion. As a result, these species are routinely found in probiotic supplements and fermented foods that attempt to improve gut health.

*Enterococci* can also become opportunistic pathogens capable of causing infections. Indeed, two of the most frequently identified species of *Enterococci – E. faecium* and *E. faecalis* – are both known to acquire antibiotic resistance. However, some strains of *E. faecium* have also been found to positively impact human health. For example, enrichment of *E. faecium* has been associated with an improved response to various types of cancer immunotherapy (Matson et al., 2018; Routy et al., 2018). Presence of these bacteria has also been shown to help prevent infections in the gut, and *E. faecium* are widely used as safe probiotics (Bhardwaj et al., 2010; Ishibashi et al., 2012).

*E. faecium* protects the gut from infections by releasing a hydrolase enzyme called secreted antigen A (SagA), which is involved in remodeling its cell wall. SagA breaks down peptidoglycans, the main component of the *E. faecium* cell wall, to produce small fragments called muramyldipeptides (MDPs; Kim et al., 2019). These MDPs activate receptors known as NOD2 in the host’s immune cells, leading to improved immunity in the gut.

Recent studies in mouse models showed that the MDPs generated by SagA could also increase anti-tumor immunity and improve cancer immunotherapy outcomes (Griffin et al., 2021). However, the role SagA plays in the bacteria itself remained unknown as it was believed that *E. faecium* needed this protein to survive. Now, in eLife, Howard Hang and colleagues – including Steven Klupt, Kyong Tkhe Fam and Xing Zhang as joint first authors – report that SagA does not affect the viability of *E. faecium,* but is required for cell wall remodeling and cell separation during replication (Klupt et al., 2024).

The team (who are based at Scripps Research) genetically modified *E. faecium* to generate a strain in which the gene for SagA was deleted. The growth of this bacterial strain was then compared to: (i) a wild-type strain, (ii) a mutant strain with inactive SagA, and (iii) a ‘complementation’ strain in which the gene for SagA had first been deleted and then re-expressed. Bacteria that lacked the gene for SagA or had an inactive version of the enzyme grew more slowly in liquid culture than wild-type *E. faecium*; but this was restored in the complementation strain. Experiments also showed that deleting the gene for SagA made *E. faecium* more sensitive to various antibiotics that target the bacterial cell wall. These exciting results raise the possibility of targeting SagA and other peptidoglycan hydrolase enzymes to overcome antibiotic resistance.

Transmission electron microscopy (TEM) – which uses a beam of electrons to generate images with ultrahigh resolution – revealed that *E. faecium* strains lacking the gene for SagA had more difficulty separating during replication. This caused the bacteria to cluster together, which could impair their growth. Cryo-electron tomography – a modification of TEM which can create three dimensional images of cells – was then used to quantify cell morphology parameters such as thickness of the cell wall and septum (a transient structure which helps to separate dividing cells). This revealed that deleting the gene for SagA alters the placement and projection angle of the new cell wall; however, this morphology was restored in the complementation strain.

To investigate the functional implications of deleting the gene for SagA, Klupt et al. used mass spectrometry to analyze specific components of the cell wall. They found that the strain in which the gene for SagA has been deleted generated fewer MDPs than wild-type *E. faecium*, which led to poor NOD2 signaling. Mouse models of cancer also did not respond to immunotherapy when they were colonized with the deficient strain. Finally, Klupt et al. demonstrated that deleting the gene for SagA reduced the population of cancer-targeting immune cells within the tumor.

Taken together, the findings show that catalytically active SagA is required for cell wall remodeling and cell separation in *E. faecium,* and its production of MDPs is required to mount an effective anti-tumor immune response (Figure 1). Furthermore, deleting the gene that codes for SagA impairs bacterial growth and increases sensitivity to antibiotics. Similar observations have been made in other bacteria that express peptidoglycan hydrolase enzymes, albeit via different mechanisms (Frirdich and Gaynor, 2013). This suggests that these enzymes could potentially be important antibacterial targets.

**Figure 1.**
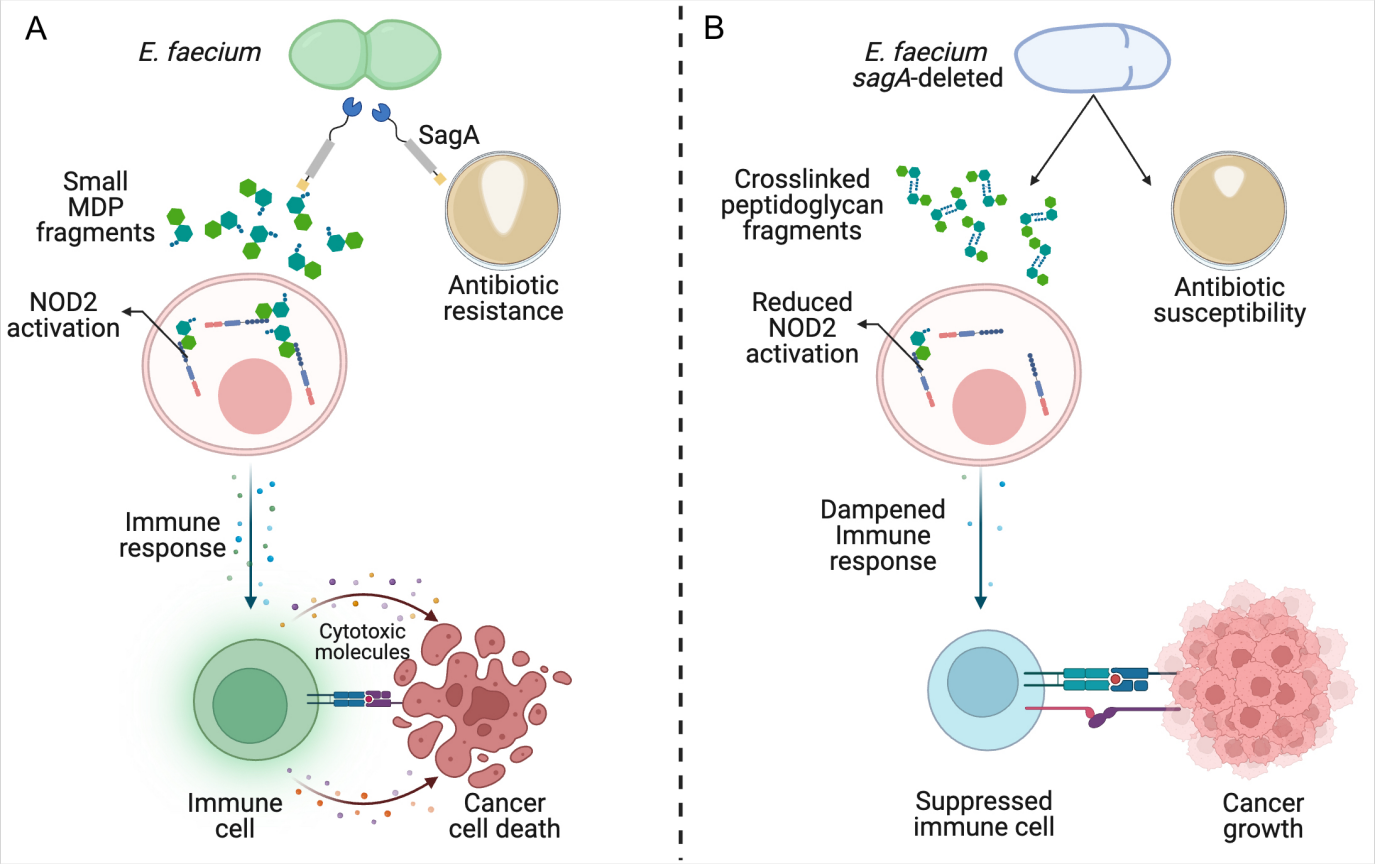
Reduced remodeling of bacterial cell walls impacts the immune response to cancer. (**A**) SagA is an enzyme that helps to remodel the cell wall of *E. faecium* by breaking down its main component, peptidoglycan. This makes the bacteria more likely to become resistant to antibiotics (brown circle; right), allowing them to grow and form larger colonies (white shape within brown circle). SagA breaks the peptidoglycan layer into small fragments called muramyldipeptides (MDP; green hexagons), which activate NOD2 receptors in the host’s immune cells (pink). This improves the outcomes of cancer immunotherapy by triggering other cells in the immune system (green) to recognize cancer cells through receptors on the cell surface (blue and purple rectangles) and release inflammatory cytotoxic molecules that will kill them. (**B**) Deleting the gene for SagA (blue cell) impairs peptidoglycan remodeling and cell separation during cell division and increases the susceptibility of *E. faecium* to antibiotics that target the cell wall, resulting in less bacterial growth and smaller colonies. The reduced cell wall remodeling results in peptidoglycan fragments remaining crosslinked, making them too large to potently activate NOD2. This lack of sufficient NOD2 signaling prevents the immune system from mounting an appropriate immune response, leading to poorer outcomes from cancer immunotherapy.

Notably, hydrolases from other immunotherapy-promoting *Enterococcus* species share more than 90% sequence homology in their catalytic domain. Future studies investigating how bacteria regulate the activity of these hydrolases could lead to better treatments for cancer and combating antibiotic resistance.
